# Low pathogenic avian influenza (H7N6) virus causing an outbreak in commercial Turkey farms in Chile

**DOI:** 10.1080/22221751.2019.1595162

**Published:** 2019-03-29

**Authors:** Pedro Jimenez-Bluhm, Nicolas Bravo-Vasquez, Mia K. Torchetti, Mary L. Killian, Brandi Livingston, Jose Herrera, Mauricio Fuentes, Stacey Schultz-Cherry, Christopher Hamilton-West

**Affiliations:** a Department of Preventive Veterinary Medicine, Faculty of Veterinary Sciences, Universidad de Chile, Santiago, Chile; b St. Jude Children’s Hospital, Memphis, TN, USA; c National Veterinary Services Laboratories, Ames, IA, USA; d Servicio Agrícola y Ganadero, Santiago, Chile

**Keywords:** Influenza, South America, Turkey, poultry, avian influenza, Chile

## Abstract

In late 2016, an H7N6 low pathogenic avian influenza virus outbreak occurred in domestic turkeys in Central Chile. We characterized the genetic and antigenic properties of the outbreak virus and its experimental transmission in chickens. Our studies demonstrate that the outbreak virus is a reassortment of genes identified from Chilean wild bird viruses between 2013 and 2017 and displays molecular adaptations to poultry and antiviral resistance to adamantanes. Further, these wild bird viruses are also able to transmit in experimentally infected chickens highlighting the need for continued surveillance and improvement of biosecurity in poultry farms.

## Introduction

Avian Influenza refers to the disease of birds caused by infection with influenza Alphaviruses (IAV) [1]. A wide variety of bird species are susceptible to IAV, including poultry and wild birds. Mainly waterfowl, gulls and shorebirds are considered the main reservoir of the virus [2,3]. The disease can be divided into two pathotypes based on its virulence and clinical presentation in poultry: highly pathogenic and low pathogenic avian influenza (HPAI and LPAI, respectively). Highly pathogenic avian influenza is characterized by a systemic disease with very high morbidity and mortality rates that can reach up to 100%, with clinical signs that are mainly respiratory [4]. On the other hand, LPAI causes a milder disease that usually has a subclinical presentation. The only IAV subtypes known to cause HPAI so far, and thus, are of mandatory notification to the World Organization for Animal Health are H5 and H7, however, these subtypes can also be of low pathogenicity [1,2,5].

**Figure 1.**
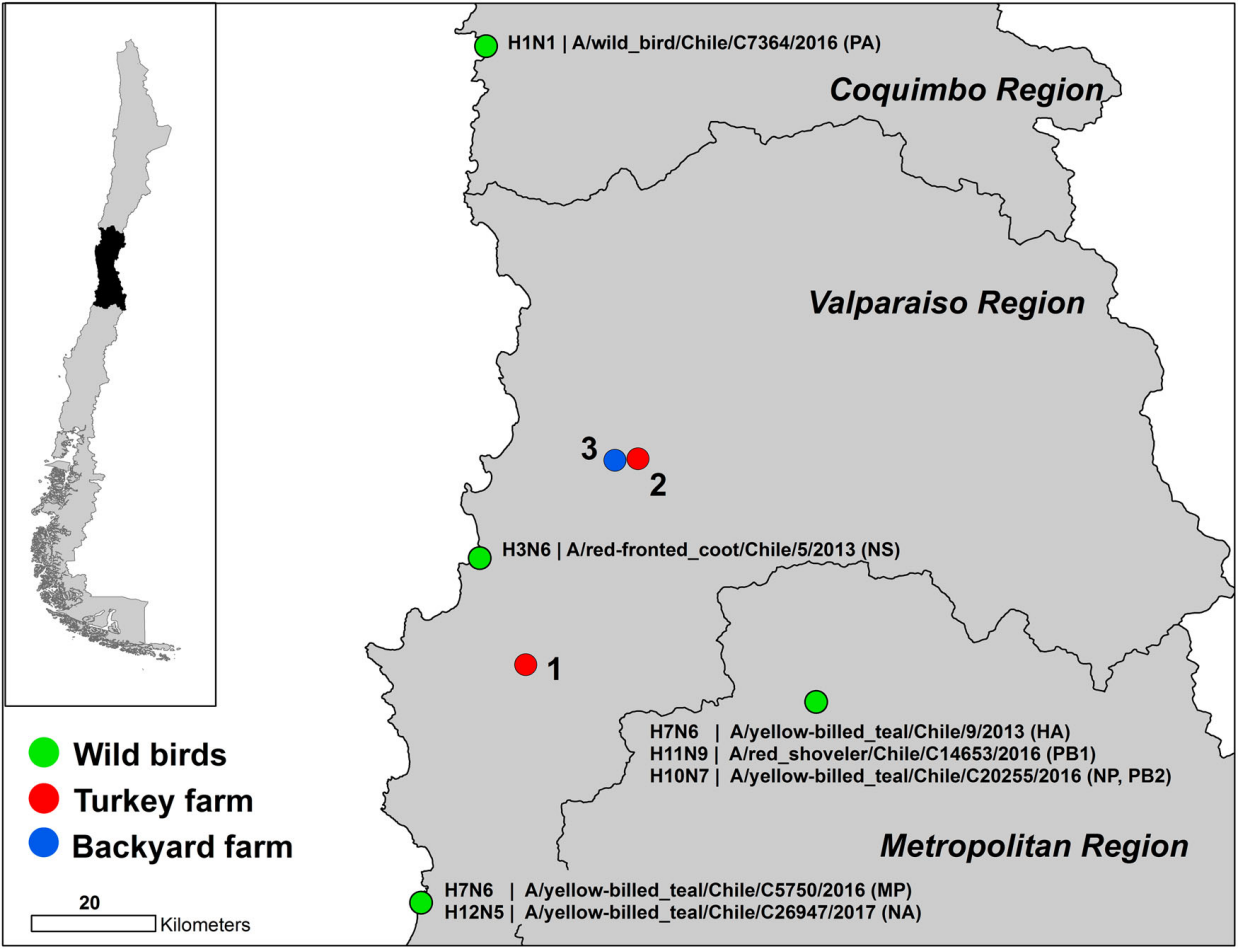
Map of the H7N6 outbreak in turkey farms in the central region of Chile. Red circles indicate the two affected farms. The blue circle shows the location of the positive backyard farm depopulated SAG. Green circles indicate location, species and genes of origin viruses.

From late December 2016 to February 2017, two outbreaks of LPAI occurred at two major turkey-fattening farms in central Chile [6]. The first report on December 26, 2016 was based on the detection of respiratory distress and an increase in mortality rates (reported mortality during the outbreak was 1.6%, while normal weekly mortality in turkey farms is 0.56%). The affected farm contained 344,540 turkeys in 62 houses. The Official Veterinary Service of Chile (SAG) diagnosed LPAI H7Nx virus using serological and RT-qPCR tests; a diagnosis that was confirmed on January 6, 2017 by the National Veterinary Services Laboratories of the US Department of Agriculture. The turkeys were immediately culled upon confirmation. On January 18, a second fattening-turkey farm, belonging to the same company, exhibited respiratory signs and increased mortality. One hundred and three of the 35,472 turkeys kept in the second farm tested positive and all animals were culled. Full genome sequencing confirmed that both outbreaks were due to LPAI H7N6 virus. On February 7, a third outbreak was reported in a backyard farm housing 425 poultry, primarily chickens with some ducks, geese and turkeys, located in the control zone of the second outbreak (Figure 1). Five of these animals tested positive by agar gel immunodiffusion (AGID) assay but remained RT-qPCR negative. The farm owner had machinery used for the removal of land for disposal of animals culled due to the outbreak. Thus, SAG required culling of all poultry from the backyard farm as a preventative measure. Chile declared itself free of avian influenza virus on June 9, 2017 [6]. In these studies, we identify the wild-bird origin of the outbreak virus and demonstrate the transmissibility of these wild-bird viruses in chicken.

## Results

### The Turkey H7N6 virus was of wild bird origin

**Figure 2.**
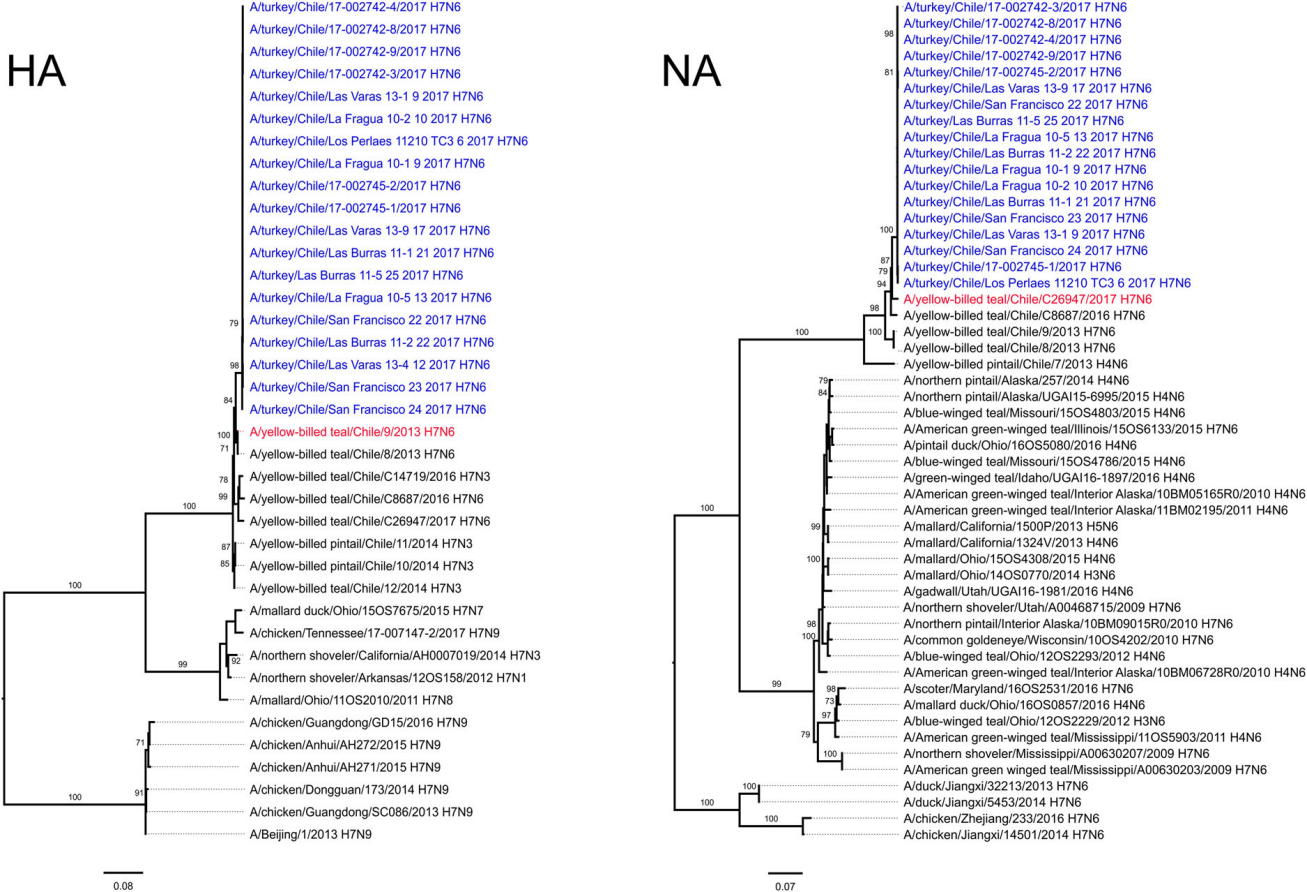
Maximum-likelihood phylogenic analysis of the HA and NA gene segments. Bootstrap values ≥70 indicated. Scale bars indicate average nucleotide substitutions per site. Outbreak viruses in blue, closest wild bird virus as established by pairwise sequence analysis (Table 1) in red.

Whole genome sequencing of 19 viruses obtained during the H7N6 LPAI outbreak showed 99.8% to 99.9% nucleotide identity between all gene segments highlighting a single introduction that spread (Table 1). Maximum-likelihood analysis (ML), coupled with pairwise sequence analysis, highlighted the close relationship amongst the outbreak viruses and their similarity to viruses of typical South American origin, obtained from wild-birds in the central region of Chile between 2013 and 2017 (Figure 2, Table 1, Supplemental Figure S1) [7]. The genome of the outbreak strain contained genes similar to genes from seven distinct wild bird viruses; A/red-fronted coot/Chile/5/2013 (NS), A/yellow-billed teal/Chile/C5750/2016 (MP), A/yellow-billed teal/Chile/C26947/2017 (NA), A/yellow-billed teal/Chile/C20255/2016 (NP), A/yellow-billed teal/Chile/9/2013 (HA), A/wild bird/Chile/C7364/2016 (PA), A/red shoveler/Chile/C14653/2016 (PB1), A/yellow-billed teal/Chile/C20255/2016 (PB2) (Figure 2, Table 1, Supplementary Figure 1).

**Table 1.** Average nucleotide identity between gene segment of outbreak isolates and estimates of evolutionary divergence to closest wild bird viruses.

Segment	Average nucleotide identity (%)	Closest sequence	Distance^a^
**NS**	99.86	A/red-fronted coot/Chile/5/2013 (H3N6)	0.01856
**MP**	99.80	A/yellow-billed teal/Chile/C5750/2016(H12N5)	0.01362
**NA**	99.92	A/yellow-billed teal/Chile/C26947/2017 (H7N6)	0.02331
**NP**	99.92	A/yellow-billed teal/Chile/C20255/2016 (H10N7)	0.00882
**HA**	99.77	A/yellow-billed teal/Chile/9/2013 (H7N6)	0.01792
**PA**	99.85	A/wild bird/Chile/C7364/2016 (H1N1)	0.01434
**PB1**	99.87	A/red shoveler/Chile/C14653/2016 (H11N9)	0.00621
**PB2**	99.79	A/yellow-billed teal/Chile/C20255/2016 (H10N7)	0.00709

^a^The number of base substitutions per site from between sequences.

Genetically, the hemagglutinin (HA) gene was similar (93% nucleotide identity) to the H7 virus responsible for a 2002 highly pathogenic avian influenza (HPAI) outbreak in central Chile and closely related to H7 viruses isolated from Chilean dabbling ducks in 2013 [7–9]. The deduced amino acid sequence of the HA cleavage site of the turkey isolates, PEKPRT**R**/GLF, was also consistent with H7 LPAI strains circulating in Chile [7,10]. According to Bayesian analysis, the estimated time to the most recent common ancestor (tMRCA) to the outbreak HA was December 9th 2011 (Bayesian credible interval: February 6^th^ 2011 to October 1^th^ 2012; posterior probability of 1) (Supplemental Figure S2).

### Genetic analysis revealed poultry adaptations and antiviral resistance

In terms of other genetic signatures, stalk deletions were observed between amino acids 38 and 63 of the neuraminidase (NA) of the turkey outbreak isolates (Supplemental Figure S3). NA stalk deletions are associated with increased adaptation to poultry and highlight the fast adaptation of this virus to Galliformes [11]. A valine-to-isoleucine substitution at amino acid 27 (V27I) was also found in the matrix protein 2 of all turkey isolates (Supplemental Figure S4). This mutation is associated with adamantane resistance [12].

### The Turkey H7N6 virus was antigenically similar to other viruses obtained in South America

Antigenically, the H7N6 LPAI outbreak viruses cross-reacted with ferret antisera generated to Chilean H7 viruses A/yellow-billed teal/Chile/8/2013 H7N6 (A/YBT/8/13) and A/yellow-billed teal/Chile/10/2014 H7N3 (A/YBT/10/14) with titers ranging from 40 to 80 (Table 2). No cross-reactivity was observed with North American H7 viruses. This confirms that Chilean viruses are antigenically and genetically distinct from North American clade H7 viruses.

**Table 2.** Hemagglutination inhibition test. Outbreak viruses A/turkey/Chile/17-002745-2/2017 and A/turkey/Chile/17-002745-2/2017 in boldface. Homologous sera inhibition values in boldface and underlined.

Reference Viruses		αYBT/Chile/2013	αYBT/Chile/2014
A/yellow-billed teal/Chile/8/2013	H7N6	**1:80**	1:80
A/yellow-billed pintail/Chile/10/2014	H7N3	1:320	**1:160**
Test Viruses			
**A/turkey/Chile/17-002745-1/2017**	H7N6	**1:80**	**1:40**
**A/turkey/Chile/17-002745-2/2017**	H7N6	**1:80**	**1:40**
A/yellow-billed teal/Chile/9/2013	H7N6	1:80	1:80
A/yellow-billed pintail/Chile/11/2014	H7N3	1:80	1:40
A/yellow-billed teal/Chile/12/2014	H7N3	1:40	1:40
A/yellow-billed teal /Chile/C8687/2016	H7N6	1:80	1:80
A/yellow-billed teal /Chile/C14719/2016	H7N3	1:40	1:40
A/duck/Alberta/48/1976	H7N3	<1:10	<1:10
A/ruddy turnstone/Delaware/270/2006	H7N3	<1:10	<1:10

Note: (α) homologous sera.

### The Turkey H7N6 virus and H7 viruses isolated from Chilean wild birds transmit in experimentally infected chicken

Finally, we evaluated the transmissibility of H7 viruses isolated from Chilean wild-birds as compared to the turkey outbreak virus in six-week-old chickens. After three days post infection (dpi), each experimental group had at least one donor chicken shedding virus via the oropharynx or cloaca routes (Supplemental Table S1). During the course of the infection the viral shedding patterns changed, showing mixed oropharyngeal and cloacal shedding until 5–7 dpi followed by mainly cloacal viral shedding after 9 dpi. Interestingly, groups infected with A/yellow-billed pintail/Chile/11/2014 H7N3 9 (A/YBT/11/14) and A/yellow-billed teal/Chile/C8687/2016 H7N6 (A/YBT/8687/16) viruses showed the highest level of infection and transmission (3/3 donors and 8/9 direct contact; 3/3 donors and 5/9 direct contact, respectively). By the end of the experiment, all chickens of the A/YBT/11/14 group showed some level of infection by EID_50_ titers matching with HAIs sera results (excepting direct contact 324, which only showed low HAI titers suggesting exposure). A similar situation occurred within the group infected with A/YBT/8687/16 although concordance between viral shedding and seroconversion is lower. Conversely, the A/turkey/Chile/17-002745-1/2017 H7N6 (A/Tk/17) group showed only two donors shedding virus by day three and five post infection and three donors with some level of seroconversion. No direct contacts were shedding virus during the course of the experiment as measured by EID_50_. Data obtained from this compared transmission experiment suggests that wild bird origin A/YBT/11/14 and A/YBT/8687/16 viruses infects and transmit better than A/Tk/17.

Additional detailed information about the obtained sequences can be found in Supplemental Table S2.

## Discussion

Avian influenza outbreaks in poultry in South America have seldomly been described, with only one HPAI outbreak registered in the continent to date, precisely in Chile in 2002 [9]. Moreover, few studies have been conducted in the region to understand the circulation of avian influenza in wild birds, hence very little is known about influenza strains circulating in bird populations in South America [7,13–15]. Our study established the wild bird origin of the H7N6 virus associated with an outbreak in domestic turkeys in central Chile. This is the second most important outbreak in terms of economic and bird losses ever registered in the continent, hence the importance of the identification of the origin of the outbreak as well as the characterization of the outbreak virus.

Epidemiologically, the genetic similarities and close timing of the outbreaks at both turkey farms suggest that the outbreak was most likely linked through movement of either birds or machinery between farms. It is also possible that backyard poultry in the control zone could have acted as an undetected reservoir of the virus and helped spread the virus to the second fattening farm. However, neither viruses nor sequences could be recovered from backyard poultry to prove this hypothesis. Previous surveillance results carried out in backyard poultry and live animal markets in Chile and Colombia have already demonstrated the circulation of wild bird viruses in domestic poultry kept under low biosecurity conditions, indicating that these systems could act as a gateway for the dissemination of wild bird-origin viruses into mainstream poultry production in South America[ 16,17].

Genetically, all gene segments originated from different wild bird origin viruses, highlighting the high reassortment rate of gene segments. This transient genome arrangement of Chilean wild bird viruses is similar to what has been described in North America and shows that avian influenza reassortment dynamics are similar across hemispheres [18]. Interestingly, the five-year gap to the tMRCA of the HA, suggests that the outbreak HA is not a direct descendant of the H7 viruses circulating in wild birds in central Chile at the time of the outbreak, but possibly an introduction from another part of the country or South America. Similarly, the putative origin of the HA of the 2002 H7N3 HPAI outbreak in Chile was a virus collected from a wild duck in Bolivia in 2001 and the HA of a LPAI H11 virus obtained at a live animal market in Colombia in 2015 was closest related to H11 viruses circulating in wild birds in Chile in 2013 [9,17]. Despite active avian influenza virus surveillance efforts made in the past 5 years in Chile, most of the surveillance efforts have been focused in the central region of Chile [7,16,19]. Therefore, only a fraction of the diversity of IAV circulating in wild birds in the country has been described. Growing evidence that South American lineage viruses can pose a serious risk to commercial poultry production is not to be underestimated, since Chile is home to 13 interhemispheric bird species that could carry potentially pathogenic IAV strains trough large geographic distances [20].

In terms of markers of pathogenicity, the NA stalk deletion found in in 18 of the 19 outbreak viruses may indicate that this virus was rapidly acquiring enhanced replication and virulence in poultry. However, the absence of increased HA glycosylation shows that the strain was still in an early stage of adaptation. Furthermore, the adamantane resistance associated V27I substitution of viral M2 protein was not present in the closely related wild-bird sequences, suggesting that it could have arisen during the outbreak together with NA truncation [21,22].

Here, we also demonstrated that H7 viruses (A/YBT/11/14 and A/YBT/8687/16) circulating in wild-birds can infect and transmit in chickens, posing a risk to poultry populations. The fact that during the transmission experiment the wild bird viruses were transmitted more efficiently than A/Tk/17, could be due the known susceptibility of turkeys to wild bird origin virus or that the genome arrangement of this virus was less capable of efficiently replicate in this host compared to wild-type viruses [23,24]. This is of concern, since the presence of influenza viruses in wild birds and its subsequent spill over into poultry has serious repercussions on animal health, public health and trade of live poultry or poultry products due to some avian influenza virus’s zoonotic potential. Further monitoring of both wild birds and domestic poultry, continuous revision and improvement of biosecurity protocols and preparedness plans in poultry farms are warranted.

## Materials and methods

### Ethics statement

All sampling activities and animal experiments were approved by the St Jude Children’s Research Hospital Institutional Animal Care and Use Committee (IACUC).

### Sequencing, genetic and phylogenic analysis

Complete genomes of 19 low pathogenic avian influenza viruses were obtained by RNA extraction, multisegment PCR and Next-gen sequencing at the National Veterinary Service Laboratory (NVSL) at Ames, Iowa, on an Illumina Miseq system using the Nextera-XT DNA Sample Preparation Kit and using the 300 cycle MiSeq Reagent Kit v2 (Illumina), as per the laboratory’s standard procedures [25]. Nucleotide sequences are available in GenBank under accession numbers MK045477 to MK045460.

Manual sequence inspection and edition of all genes was performed using BioEdit [26]. Putative glycosylation sites were identified by Asn-X-Ser/Thr motives from the consensus sequence of the outbreak HA. Sequence alignment was performed using MUSCLE [27]. Reference sequences were obtained from the Influenza Virus Resource site from NCBI [28]. Representative sequences from Asia and North America where chosen for the analysis as well as all available wild bird origin sequences from Chile, with the exception to seabird associated H13 viruses. Additional North American sequences for the ML analysis of the NA (n = 20) and the Bayesian analysis of the HA (n = 20) where selected for each dataset by random selection on an excel spreadsheet. This process was repeated twice to assure consistency of groups. Finally, the Bayesian analysis of the HA also included sequences of the Chilean HPAI 2002 outbreak (n = 9). ModelTest was used in order to establish the best-fit nucleotide substitution model in MEGA7 [29]. A maximum-likelihood phylogenic analysis for each gene using RAxML with a GTR + G nucleotide substitution model was performed [30]. In order to test for the robustness of the trees, one thousand bootstrap replicates where run. Additionally, pairwise genetic distances of the alignments were calculated using the Tamura-Nei substitution Model with a gamma distribution in Mega7. ML phylogenic inferences were inspected with TempEst to identify sequence outliers [31]. After removing these, a time-scaled phylogenic analysis using BEAST [32] was performed. An HKY + G nucleotide substitution model with a Bayesian lognormal relaxed-clock Markov chain Monte Carlo (MCMC) method was applied to sample trees. For each analysis a Bayesian Skyline coalescent tree prior model was used (10 groups). The starting tree was chosen randomly and three independent analyses of 50 million generations each and sampling every 15000 generations was carried out. Parameters where assessed for convergence with Tracer for an effective sample size (ESS) of >200. 10% of the burn-ins were removed and the independent chains were merged using LogCombiner. Trees and parameters were summarized with TreeAnnotator and the maximum clade credibility (MMC) tree was visualized using FigTree.

### Serological analysis

Nine H7 LPAI viruses isolated from Chilean wild birds (A/yellow-billed teal/Chile/8/2013 H7N6 ; A/yellow-billed teal/Chile/9/2013 H7N6; A/yellow-billed pintail/Chile/10/2014 H7N3; A/yellow-billed pintail/Chile/11/2014 H7N3; A/yellow-billed teal/Chile/12/2014 H7N3; A/ yellow-billed teal /Chile/C8687/2016 H7N6 and A/yellow-billed teal /Chile/C14719/2016 H7N3) and commercial poultry farms (A/turkey/Chile/17-002745-1/2017 H7N6 and A/turkey/Chile/17-002745-2/2017 H7N6) as well as two North American origin wild bird viruses (A/duck/Alberta/48/1976 H7N3; A/ruddy turnstone/Delaware/270/2006 H7N3) were tested in an hemagglutination inhibition (HAI) assay against a panel of two ferret antisera (A/yellow-billed pintail/Chile/8/2013 H7N6 and A/yellow-billed pintail/Chile/10/2014 H7N3). Briefly, 25 µL of sera treated with a receptor destroying enzyme (RDE) (cat: 370013, Denka Seiken, Co., Ltd.) were 2-fold serially diluted in 25 µL of PBS in duplicate in a 96 v-bottom well plate. Twenty-five µL of a solution containing 4 hemagglutinin units (HAU) of each virus were added and incubated for 15 min. Finally, 50 µL of 0.05% chicken red blood cells were added and the plate was placed at 4°C for 30–45 min after which the assay was read.

### Chicken transmission experiment

Three days old SPF white leghorn embryonated chicken eggs (cat: 101000329, Charles River Laboratories Inc.) were incubated for 21 days until hatched. Chicks were transferred to an ABL2 facility by 6 weeks of age and divided into five experimental infection groups (A/yellow-billed teal/Chile/9/2013 H7N6; A/yellow-billed pintail/Chile/11/2014 H7N3; A/yellow-billed teal/Chile/C14719/2016 H7N3; A/yellow-billed teal/Chile/C8687/2016 H7N6 and A/turkey/Chile/17-002745-1/2017 H7N6) and one PBS control group. For each experimental group, three donors and nine direct contact chickens were settled into three cages inside isolated cubicles. Separately, donors were infected through natural route (eyes, nares and trachea) with 10^6^ EID_50_/0.5 mL and transferred 24 h. later into a cage with its corresponding direct contacts. The inoculum was checked by EID_50_ before and after the infection. All chickens were observed during the course of the infection to check illness symptomatology. Oropharyngeal and cloacal swabs were collected in a 1:1 glycerol/PBS plus antibiotics media (Penicillin 1000 units/mL; Streptomycin 200 µg/mL; Nystatin (Mycostatin) 50 units/mL; Gentamicin 250 μg/mL and Polymixin B 100 units/mL) at 3, 5, 7, 9- and 12-days post infection (dpi) and stored at −80°C for further analysis [33]. Finally, 1 mL of blood was collected at day 18 post infection in order to check seroconversion by performing HAI and chickens were tested with its corresponding homologous virus strain according to their assigned group. All birds were euthanized at 21 dpi. Swab media was inoculated in duplicate into 9–11 days old eggs with a 1:10 PBS dilution/antibiotics solution and viral titers were determined by the method of Reed and Munch by performing EID_50_ analysis with 50 µL allantoic fluid and 50 µL of 0.05% chicken red blood cells [34].

## Supplementary Material

Supplemental Material
