# Supplementary material for: Low pathogenic avian influenza (H7N6) virus causing an outbreak in commercial Turkey farms in Chile
Source: Emerg Microbes Infect. 2019 Mar 29;8(1):479–85. doi: 10.1080/22221751.2019.1595162 (PMC6456847; doi:10.1080/22221751.2019.1595162)

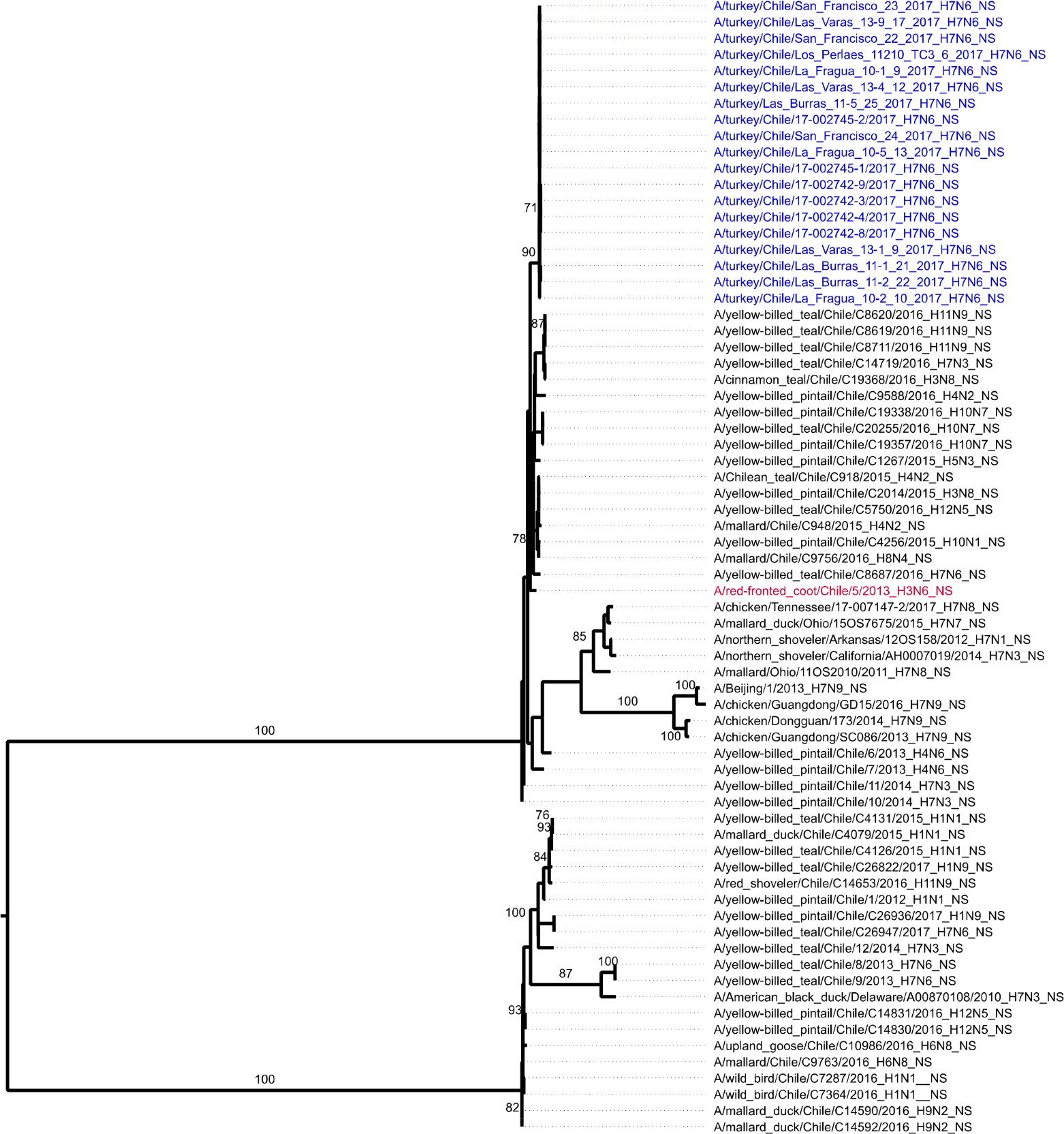


A) NS

Supplemental Figure S1. Maximum-likelihood phylogenic analysis of the H7N6 internal gene segments. A) NS, B) MP, C) NP, D) PA, E) PB1 and F) PB2. Bootstrap values ≥70 indicated. Scale bars indicate average nucleotide substitutions per site. Outbreak viruses in blue, closest putative wild bird virus as established by pairwise sequence analysis (Table 1) in red.


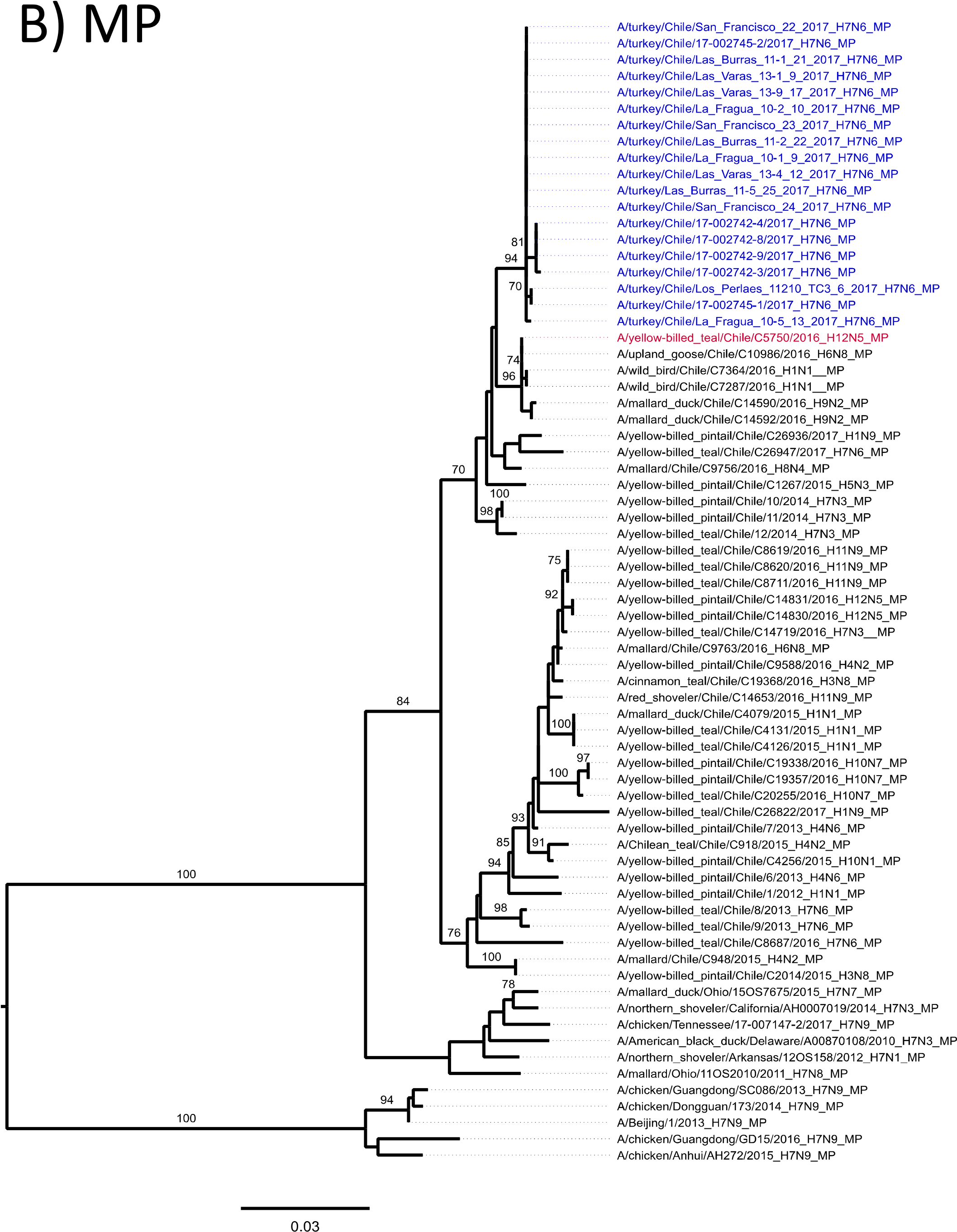


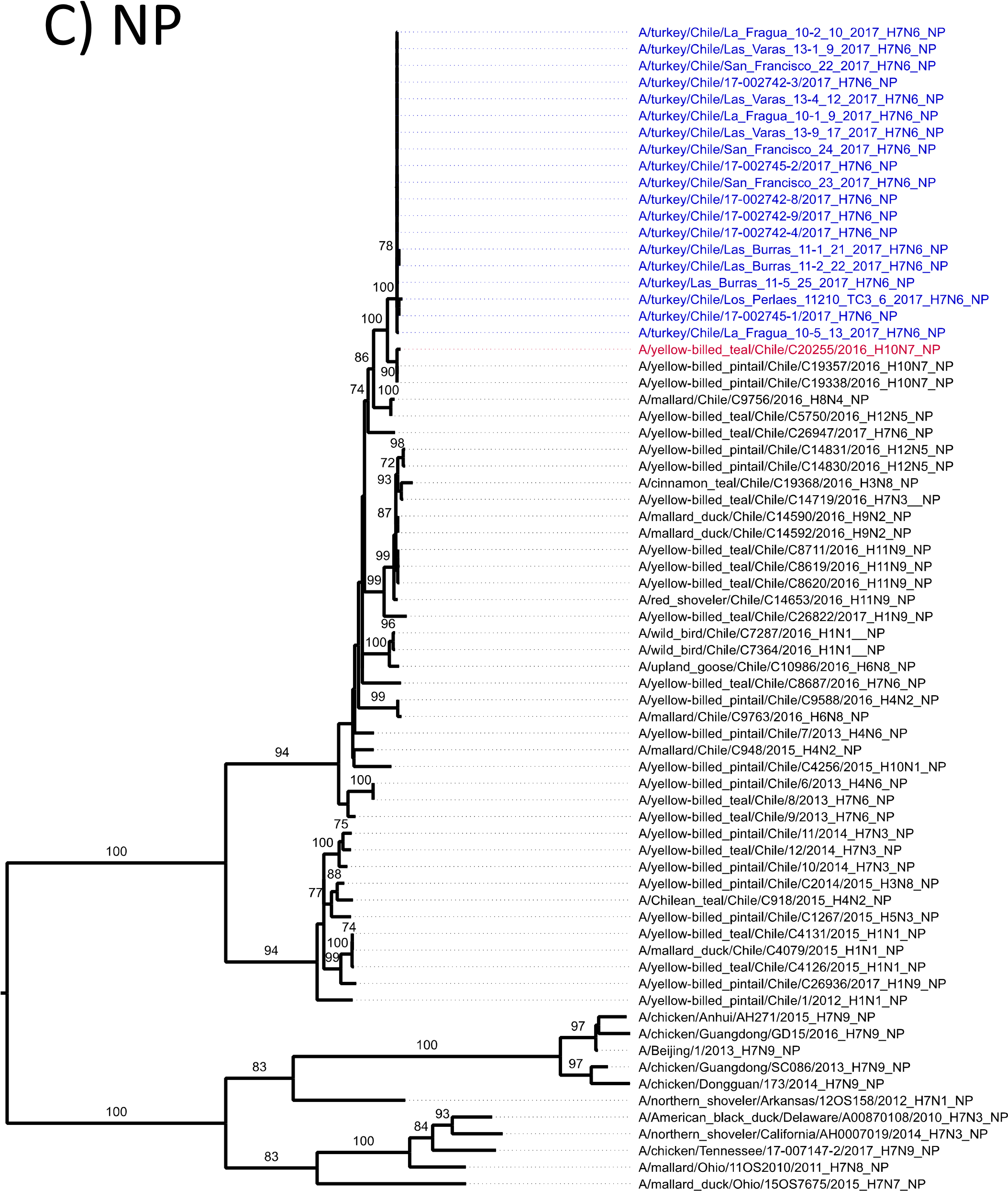


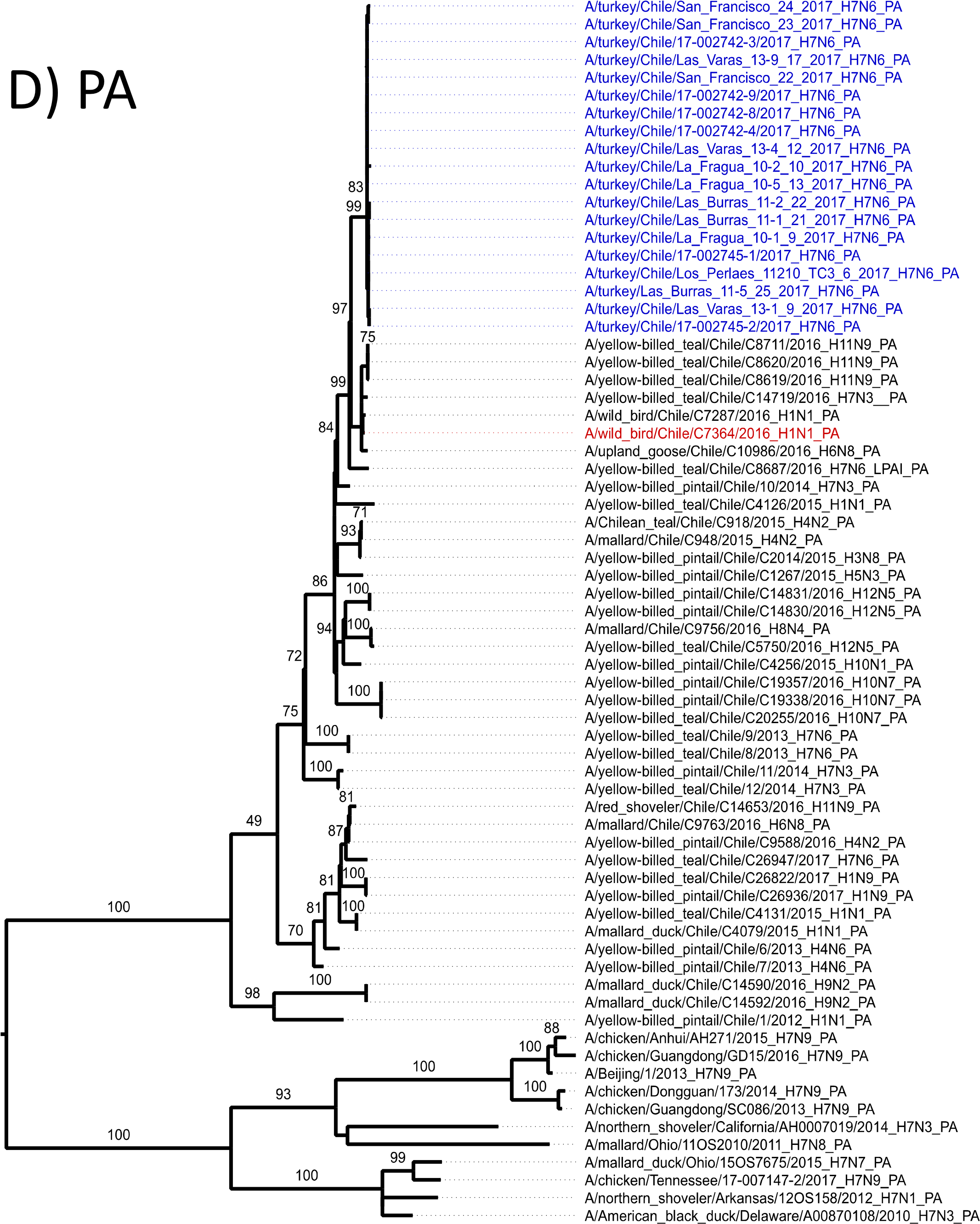


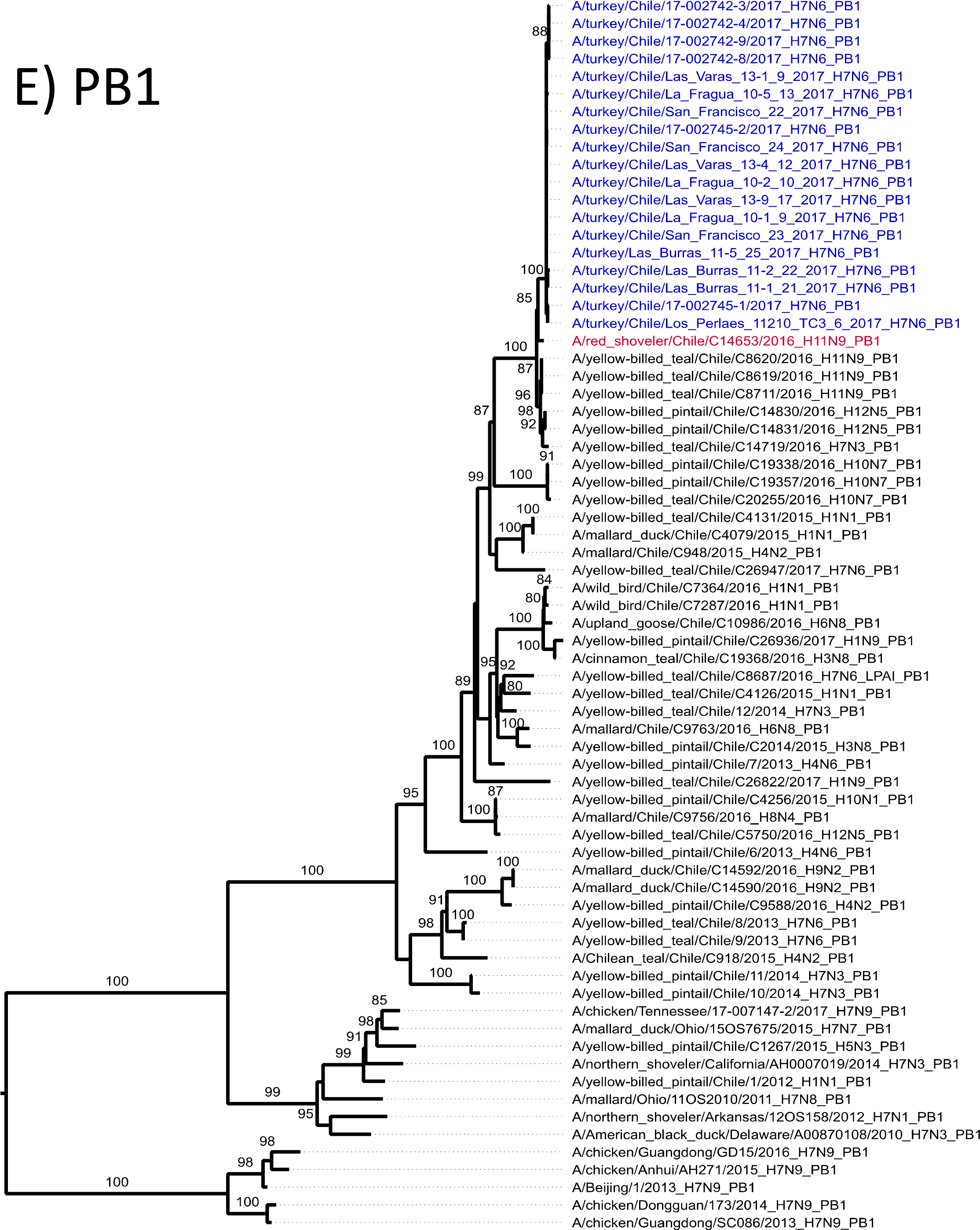


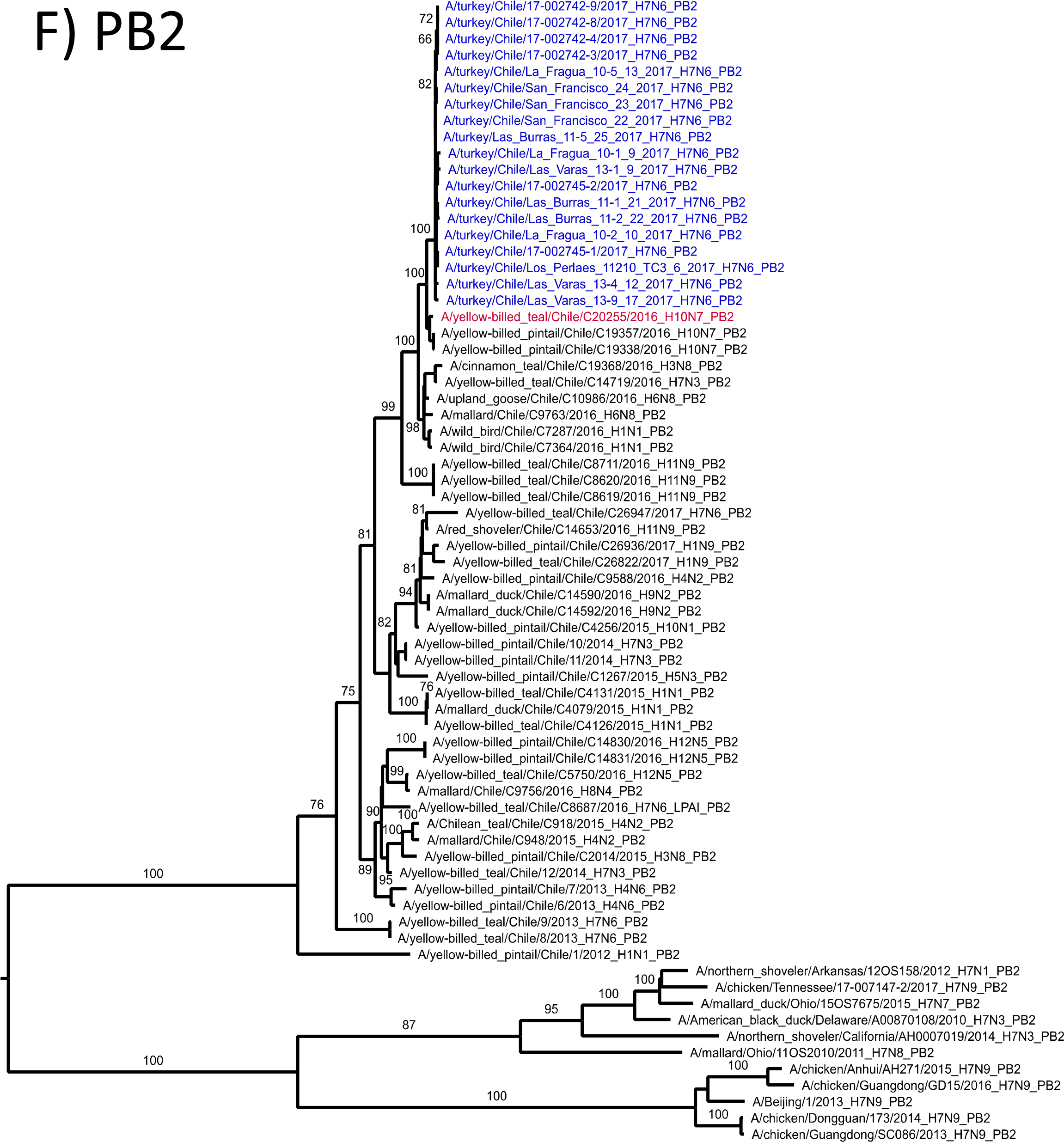

Supplement: Supplemental Material [file TEMI_A_1595162_SM1262.zip › Supplementary Material/Supplemental_Figure_S1.docx]
