# Supplementary material for: Low pathogenic avian influenza (H7N6) virus causing an outbreak in commercial Turkey farms in Chile
Source: Emerg Microbes Infect. 2019 Mar 29;8(1):479–85. doi: 10.1080/22221751.2019.1595162 (PMC6456847; doi:10.1080/22221751.2019.1595162)
Supplement: Supplemental Material [file TEMI_A_1595162_SM1262.zip › Supplementary Material/Supplemental_Figure_S2.docx]

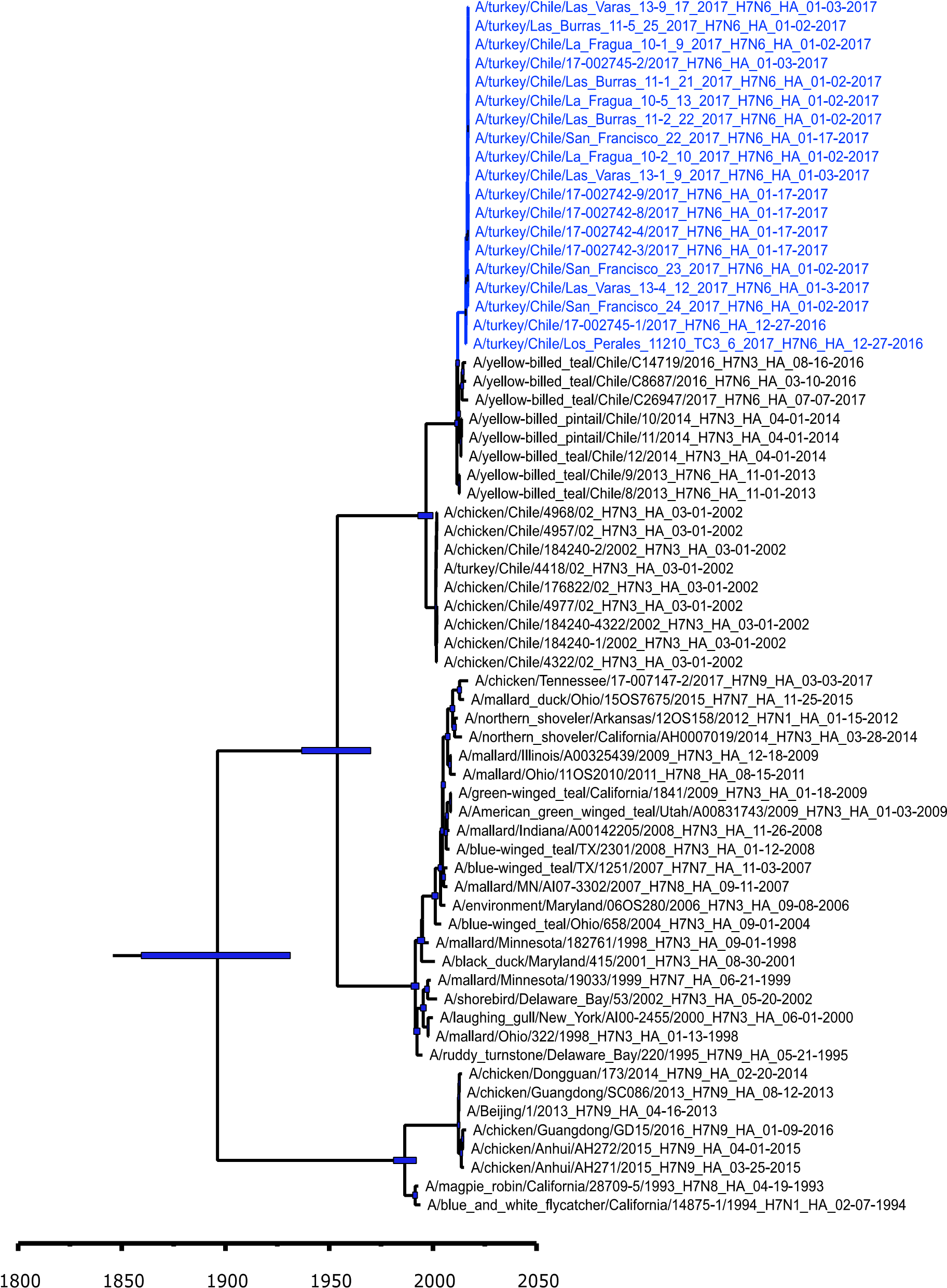


Supplemental Figure S2. Bayesian MMC tree of the H7 outbreak. Time stamped tree that shows temporal and phylogenic relationships between viruses obtained during the outbreak and other H7 viruses obtained in Chile, North America and Eurasia. Node bars indicate the 95% Bayesian Credible interval. Outbreak viruses in blue.
