# Supplementary material for: Low pathogenic avian influenza (H7N6) virus causing an outbreak in commercial Turkey farms in Chile
Source: Emerg Microbes Infect. 2019 Mar 29;8(1):479–85. doi: 10.1080/22221751.2019.1595162 (PMC6456847; doi:10.1080/22221751.2019.1595162)
Supplement: Supplemental Material [file TEMI_A_1595162_SM1262.zip › Supplementary Material/Supplemental_Figure_S3_S4.docx]

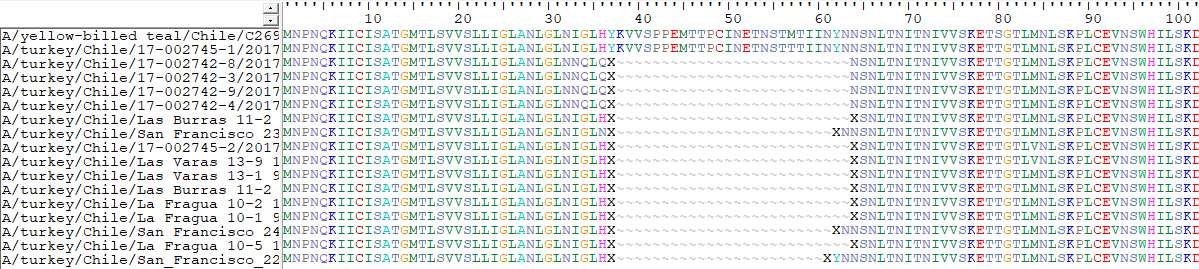

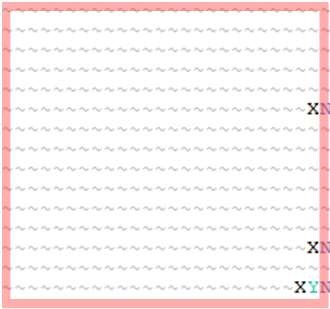


Supplemental Figure S3. NA stalk deletion position of outbreak viruses. Sequence alignment of the NA of the closest wild bird sequence A/yellow-billed teal/Chile/C26947/2017 to the H7N6 outbreak strain. Full length NA outbreak sequence A /turkey/Chile/17002745-1/2017 is also provided as reference. Red square indicates positions of amino acid deletions between amino acids 38 and 63.


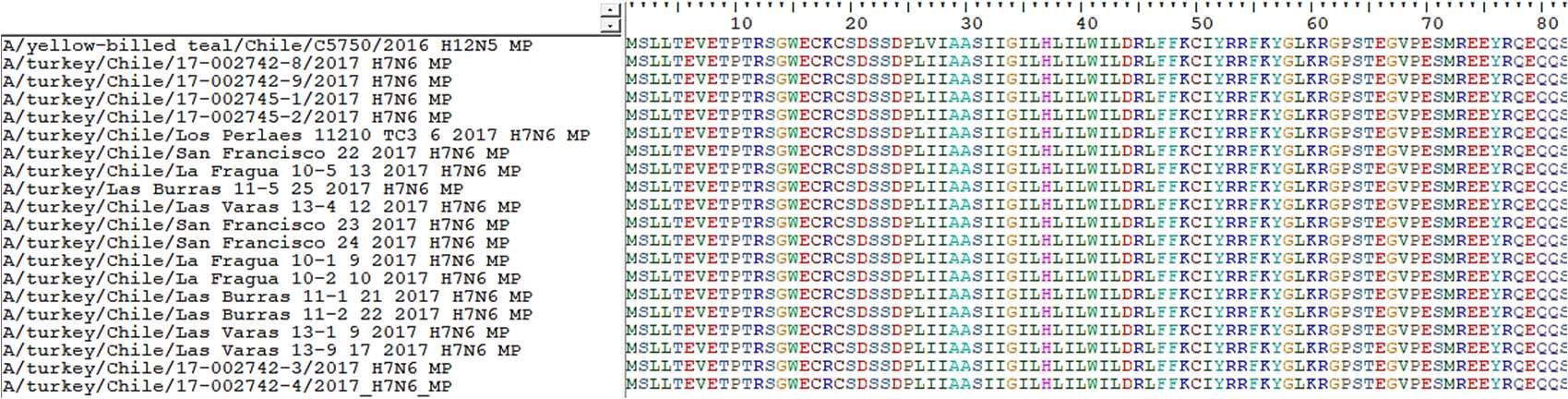


V27I

Supplemental Figure S4. Alignment analysis of the M2 sequence. Sequences of all H7N6 isolates obtained during the outbreak show amino acid substitution V27I within the transmembrane domain of M2 protein. Closest wild bird M2 sequence A/yellow-billed teal/Chile/C5750/2016 (H12N5) shown as reference does not display the substitution.
