# Supplementary material for: Low pathogenic avian influenza (H7N6) virus causing an outbreak in commercial Turkey farms in Chile
Source: Emerg Microbes Infect. 2019 Mar 29;8(1):479–85. doi: 10.1080/22221751.2019.1595162 (PMC6456847; doi:10.1080/22221751.2019.1595162)
Supplement: Supplemental Material [file TEMI_A_1595162_SM1262.zip › Supplementary Material/Supplemental_Table_S1.docx]

Supplemental Table S1. Chicken transmission study. Oropharyngeal and cloacal

swabs titers and HAI results. Yellow cell indicates donor. EID50 titer expressed as log/

ml. HAI titers against homologous test virus.

|  | ID | OP^a^ | CL^b^ | OP | CL | OP | CL | OP | CL | OP | CL | HAI^c^ |
| --- | --- | --- | --- | --- | --- | --- | --- | --- | --- | --- | --- | --- |
|  |  | 3 dpi | | 5 dpi | | 7 dpi | | 9 dpi | | 12 dpi | | 18 dpi |
| YBT/Chile/9/2013 H7N6 | 304 | -d | - | - | - | - | - | - | - | - | - | <1:10 |
|  | 307 | - | - | - | - | - | - | - | - | - | - | <1:10 |
|  | 308 | - | - | - | - | - | - | - | - | - | - | <1:10 |
|  | 309 | - | - | - | - | - | - | - | - | - | - | <1:10 |
|  | 305 | - | 7.5 | - | 8.5 | - | 2 | - | 3 | - | - | 1:40 |
|  | 310 | - | - | 3.5 | - | - | 2.4 | - | - | - | - | <1:10 |
|  | 311 | - | - | - | - | - | - | - | - | - | - | <1:10 |
|  | 312 | - | 4 | 2 | 2.5 | - | 4 | - | - | - | - | 1:40 |
|  | 306 | - | - | - | - | - | - | - | - | - | - | <1:10 |
|  | 313 | - | - | - | - | - | - | - | - | - | - | <1:10 |
|  | 314 | - | - | - | - | - | - | - | - | - | - | <1:10 |
|  | 301 | - | - | - | - | - | - | - | - | - | - | <1:10 |
| YBP/Chile/11/2014 H7N3 | 316 | 4.4 | - | - | - | - | - | - | - | - | - | 1:80 |
|  | 319 | 3.5 | - | 2.5 | 4.5 |  | 4 | - | - | - | - | 1:20 |
|  | 320 | - | - | 4 | - | 3.6 | - | - | - | - | - | 1:20 |
|  | 321 | - | - | - | 5 | 3 | 5 | - | - | - | - | 1:40 |
|  | 317 | 6.5 | - | - | - | - | - | - | - | - | - | 1:40 |
|  | 322 | - | - | - | 3 | - | 4.5 | - | 3.5 | - | 3.75 | 1:40 |
|  | 323 | - | - | - | - | - | 2.4 | - | 6.25 | - | 4.5 | 1:40 |
|  | 324 | - | - | - | - | - | - | - | - | - | - | 1:20 |
|  | 318 | 5.5 | 6.5 | 3.5 | 4.5 | - | 5.5 | - | 3.5 | - | - | 1:40 |
|  | 325 | 3.5 |  | 2.5 | 4.5 | - | 3.25 | - |  | - | - | 1:20 |
|  | 326 | 4 | 5 | 3.75 | 4.5 | - | 6.5 | - | 4.5 | - | 2.4 | 1:40 |
|  | 327 | 5 | 3.5 | 5.5 | 4.5 | - | 3 | - | - | - | - | 1:40 |
| YBT/Chile/C14719/20 16 H7N3 | 328 | 3 | 4 | 4 | 3.5 | - | 6 | - | - | - | - | 1:80 |
|  | 331 | - | 2 | 3.5 | - | - | - | - | - | - | - | 1:20 |
|  | 332 | 3 | - | - | - | - | - | - | - | - | - | <1:10 |
|  | 333 | 2 | 2.5 | 2.75 | 3.5 | - | 3.5 | - | - | - | - | 1:80 |
|  | 329 | 2.5 | - | - | - | - | - | - | - | - | - | 1:20 |
|  | 334 | - | - | - | - | - | - | - | - | - | - | <1:10 |
|  | 335 | - | - | - | - | - | - | - | - | - | - | <1:10 |
|  | 336 | - | - | - | - | - | - | - | - | - | - | 1:20 |
|  | 330 | - | - | 2.5 | 6.5 | - | 5 | - | - | - | - | 1:80 |
|  | 337 | - | - | 3 | 2 | - | 7.5 |  | 6 |  | 5 | 1:80 |
|  | 338 | - | - | 2 | - | 4 | 3.5 | 2.75 | 3.5 | - | 4.6 | 1:160 |
|  | 339 | - | - | - | - | 4.75 | 4.5 | - | 5 | - | 2.75 | 1:80 |
| YBT/Chile/C8687/ 2016 H7N6 | 340 | 3 | - | - | 4 | - | 4.5 | - | 2.4 | - | - | 1:20 |
|  | 343 | - | - | - | - | - | - | - | - | - | - | <1:10 |
|  | 344 | - | - | - | - | - | - | - | - | - | - | 1:40 |
|  | 345 | - | - | - | - | - | 2.5 | - | 2.75 | - | 5 | 1:40 |
|  | 341 | - | 4.5 | - | - | - | 3.5 | - | - | - | 3.5 | 1:40 |
|  | 346 | - | - | - | - | - | - | - | - | - | - | <1:10 |
|  | 347 | 3 | 2 | 4.5 | - | - | 2.75 | - | - | - | - | 1:20 |
|  | 348 | 2.5 | - | - | - | - | - | - | - | - | - | <1:10 |
|  | 342 | - | 3 | 2.5 | 3 | - | 3.5 | - | 2.5 | - | - | 1:80 |
|  | 349 | - | - | - | - | - | - | - | - | - | - | <1:10 |
|  | 350 | - | - | 4 | 4 | 2.75 | 5 | - | 4 | - | 4.5 | 1:20 |
|  | 351 | - | - | 2 | 5 | - | 2.5 | - | - | - | - | 1:20 |
| Turkey/Chile/17-  002745-1/2017 H7N6 | 352 | - | - | - | - | - | - | - | - | - | - | 1:20 |
|  | 355 | - | - | - | - | - | - | - | - | - | - | <1:10 |
|  | 356 | - | - | - | - | - | - | - | - | - | - | <1:10 |
|  | 357 | - | - | - | - | - | - | - | - | - | - | <1:10 |
|  | 358 | - | - | 4.5 | - | - | 4 | - | 2.75 |  | 4.5 | 1:20 |
|  | 353 | - | - | - | - | - | - | - | - | - | - | <1:10 |
|  | 359 | - | - | - | - | - | - | - | - | - | - | <1:10 |
|  | 360 | - | - | - | - | - | - | - | - | - | - | <1:10 |
|  | 354 | - | 5.5 | - | - | - | 4.75 | - | 4.5 | - | 2.4 | 1:80 |
|  | 361 | - | - | - | - | - | - | - | - | - | - | <1:10 |
|  | 362 | - | - | - | - | - | - | - | - | - | - | <1:10 |
|  | 302 | - | - | - | - | - | - | - | - | - | - | <1:10 |

^a^=oropharyngeal; ^b^= cloacal; ^c^=hemagglutination inhibition; ^d^=Log egg infectious dose 50 (EID_50_) titer
